# Aggression, self-control, life satisfaction, and resilience as predictors of mental health in Brazilian jiu-jitsu athletes

**DOI:** 10.3389/fspor.2025.1692536

**Published:** 2025-11-06

**Authors:** Leandro de Lorenco-Lima, Stacey A. Gaines, Elisabeth M. Waterbury

**Affiliations:** Department of Psychology, Liberty University, Lynchburg, VA, United States

**Keywords:** mental health disorders, combat sports, martial arts, aggression, self-Control, life satisfaction, resilience

## Abstract

**Introduction:**

Brazilian jiu-jitsu has been discussed as an effective type of psychosocial therapy, with the social interactions within the Brazilian jiu-jitsu community serving as a buffer against mental health disorders. However, the psychological variables associated with optimal mental health in Brazilian jiu-jitsu have yet to be explored. This study examined the extent to which resilience, grit, self-efficacy, self-control, aggression, and life satisfaction (IVs) could predict mental health in male and female Brazilian jiu-jitsu athletes.

**Methods:**

The sample included 420 athletes, representing 331 males (78.8%) and 89 females (21.2%) from 18 to 60 years of age (38.2 ± 8.8), who responded to training-related questions followed by the Mental Health Disorders Screening Instrument for Athletes, Brief Resilience Scale, Grit Scale, General Self-Efficacy Scale, Brief Self-Control Scale, Brief Aggression Questionnaire, and Satisfaction with Life Scale.

**Results:**

In males, results revealed that a multiple linear regression was statistically significant, with the IVs accounting for approximately 51.7% of the variance in mental health disorders. Aggression (9.1% variance), self-control (7.3% variance), life satisfaction (5.0% variance), and resilience (1.2% variance) were significantly associated with mental health disorders in male athletes. In females, a multiple linear regression was statistically significant, with the IVs accounting for approximately 45.3% of the variance in mental health disorders. Aggression (6.0% variance), and life satisfaction (3.0% variance) were significantly associated with mental health disorders in female athletes.

**Conclusion:**

In conclusion, male Brazilian jiu-jitsu athletes presenting higher self-control, life satisfaction, and resilience, and lower aggression, and female athletes presenting higher life satisfaction and lower aggression were more likely to present better mental health than athletes with opposing characteristics. With the rising popularity of Brazilian jiu-jitsu, these findings may inform clinical professionals when developing treatment plans to address mental health in athletes in this population.

## Introduction

In Western societies, the number of people affected by mental health disorders is believed to be greatly under-reported (1). The Diagnostic and Statistical Manual of Mental Disorders defines mental health disorders as “a syndrome characterized by clinically significant disturbance in an individual's cognition, emotion regulation, or behavior that reflects a dysfunction in the psychological, biological, or developmental processes underlying mental functioning” [(2), p. 14]. Mental health disorders are often associated with substantial distress or disability in daily activities, such as social and occupational tasks (2). Mental health disorders are associated with increased aggression, inattentiveness/hyperactivity, and emotional challenges (3).

In search for interventions to effectively address mental health disorder, research has shown that sports can provide substantial physical and mental health benefits in the short- and long-term (4), with some authors suggesting that their social and mental health benefits may exceed those acquired through alternative leisure activities (5, 6). As a subcategory of sports, in particular combat sports, Brazilian jiu-jitsu has gained tremendous popularity across the world over the past decades, and more recently attracted the attention of behavioral and social scientists. The dynamics of Brazilian jiu-jitsu involves two athletes aiming to subdue one another via “tap-out”. Progressively, the “tap-out” can be achieved by taking an opponent to the ground via throws, takedowns, or pulling guard, controlling the opponent, and performing submission holds such as strangles and joint locks (7). In competitions, two athletes grapple for victory with the rules determining the winner by submission, points, loss of consciousness, disqualification, or the referee's decision (8). After years of training under this ruleset, psychological difference can be found in Brazilian jiu-jitsu athletes of different experience and belt rank, as more experienced practitioners were found to present higher mental strength, resilience, grit, self-efficacy, self-control, physical and verbal aggression, and life satisfaction than less experienced practitioners (9). Moreover, Brazilian jiu-jitsu black belts reported significantly lower mental health disorders than white belts (10).

In addition to the rank-based psychological differences, Brazilian jiu-jitsu has been discussed by some authors as an effective type of psychosocial therapy (1, 11–13) and a positive intervention to address mental health disorders (13, 14). Brazilian jiu-jitsu's therapeutic characteristics are described as being the result of athletes consistently placing themselves under great psychological and physical stress, and therefore, igniting several intrinsic resources critical for developing coping skills that may lead to mental health improvements (1). Chinkov and Holt (15) reported that the engagement with Brazilian jiu-jitsu can be life-changing by fostering the acquisition of four critical life skills: respect for others, perseverance, self-confidence, and healthy habits. Moreover, Blomqvist-Mickelsson (12) explains that the social interactions with like-minded individuals in the Brazilian jiu-jitsu community may serve as a protective buffer against mental health disorders and promote well-being.

When applied as an intervention, the existing literature has found Brazilian jiu-jitsu to be effective in decreasing psychopathological symptoms (14), post-traumatic stress disorder (13, 14), aggression, and increase self-control (16). It has also been found effective in improving children's emotional symptoms, hyperactivity/inattention, total difficulties score, and externalizing problems (17). Ruelas et al. (18) presented that participants credited Brazilian jiu-jitsu for substantial improvements in mental flexibility, confidence, strengthened commitment, reduced anxiety, and a strong sense of community. Adult practitioners also experienced improvements in mood and respect, while parents noticed the acquisition of critical life skills by their children (18).

Given the rising concern with mental health worldwide, the unique Brazilian jiu-jitsu ruleset, the psycholgocial benefits associated with Brazilian jiu-jitsu training (13, 14, 17, 18), and the rank-based psychological differences among white and black belts, the present study aimed to explore the role of several psychological variables, found to be elevated in black belts, in predicting mental health disorder in Brazilian jiu-jitsu athletes. The novelty of this study is based on its first of a kind approach aiming to explore how the psychological characteristics often present in more experienced Brazilian jiu-jitsu athletes may impact mental health disorders in a population practicing a sport where vigorous combats are an inherent characteristic of the activity. With the rising popularity of Brazilian jiu-jitsu, the result of this study can inform clinical mental health professionals about critical variables associated with positive mental health outcomes and, consequently, guide the development of clinical treatment plans. Therefore, the purpose of this study was to examine the extent to which resilience, grit, self-efficacy, self-control, aggression, and life satisfaction could predict mental health disorders in Brazilian jiu-jitsu athletes.

## Methods

### Participants

Participants included 420 Brazilian jiu-jitsu athletes, 331 males (78.8%) and 89 females (21.2%), from 18 to 60 years of age (38.2 ± 8.8). Participants represented 121 white belts (28.8%), 118 blue belts (28.1%), 78 purple belts (18.6%), 46 brown belts (10.9%) and 57 black belts (13.6%). A total of 189 athletes (45%) participated in at least one competition over the previous year, and 231 athletes (55%) participated in no competition during the same period. Participants represented forty-seven states and the District of Columbia. The states with the highest number of participants were Nevada (19%), Pennsylvania (9%), and California (7.9%). This study included no participants from Rhode Island, West Virginia, or Wyoming.

### Procedures

In the current cross-sectional study, participants responded to an anonymous online survey disseminated through social media (Facebook and Instagram) via Google Forms from April 19 to June 10, 2024. Inclusion criteria: male and female, from 18 to 60 years of age, of all belt ranks (white, blue, purple, brown, and black), and engaging in a minimum of one Brazilian jiu-jitsu class per week in the United States. The Google Forms included demographic questions (age, biological sex, and state of residency), Brazilian jiu-jitsu training characteristics questions (training experience in years, belt rank, number of competitions over the previous 12 months, and number of days and hours of training per week), in addition to the Mental Health Disorders Screening Instrument for Athletes, Brief Resilience Scale, Grit Scale, General Self-Efficacy Scale, Brief Self-Control Scale, and Satisfaction with Life Scale.

This study was conducted anonymously, and no compensation was offered for participation. Volunteers received an information sheet presenting the details of the study. This study was exempted by the Liberty University Institutional Review Board, based on 45 CFR 46:104(d): Category 2. (i). clarifying that the data obtained by the author is recorded in a way that the identity of the human participants cannot readily be ascertained directly or through identifiers linked to the participants (19).

### Materials

Mental health disorder was assessed through the Mental Health Disorders Screening Instrument for Athletes (14-item, 7-point Likert) based on its good internal consistency and Cronbach's alpha of .86 (20). The sum of the 14 items determined total scores, and higher scores represented higher mental health disorders. Cronbach's alpha in the current sample was 0.82.

Resilience was assessed through the Brief Resilience Scale (6-item, 5-point Likert) based on its good internal consistency with Cronbach's alpha ranging from .80 to .91 (21). The average of the six items determined total scores, and higher scores indicated higher resilience. Cronbach's alpha in the current sample was .82.

Grit was assessed through the Grit Scale (12-item, 5-point Likert) due to its good internal consistency with Cronbach's alpha of .85 (22). The average of the 12 items determined total scores, and higher scores indicated higher grit. Cronbach's alpha in the current sample was .84.

Self-efficacy was assessed through the General Self-Efficacy Scale (10-item, 4-point Likert) based on its good internal consistency with Cronbach's alpha between .76 and .90 (23). The sum of the 10 items calculated total scores, and higher scores indicate higher self-efficacy. Cronbach's alpha in the current sample was .87.

Self-control was assessed through the Brief Self-Control Scale (13-item, 5-point Likert) due to its internal consistency with Cronbach's alpha of .83 and .85 (24). The sum of the 13 items determined total scores, and higher scores indicate higher self-control. Cronbach's alpha in the current sample was .84.

Aggression was assessed through the Brief Aggression Questionnaire (12-item, 7-point Likert) based on its strong test-retest reliability, ranging from .68 to .80 (25, 26). Total scores were calculated by the average of the 12 items, with higher scores indicating higher aggression. Cronbach's alpha in the current sample was .76.

Life satisfaction was assessed through the Satisfaction with Life Scale (5-item, 7-point Likert) due to its good internal consistency and Cronbach's alpha of .87 (27). The sum of the five items calculated total scores, and higher scores indicate higher life satisfaction. Cronbach's alpha in the current sample was .87.

### Statistical analyses

All assumptions of normality were met in the current data (skewness and kurtosis values, histograms, Q-Q plots, and Shapiro–Wilk). Descriptive data were presented as mean and standard deviation (M ± SD). As primary analyses, a multiple linear regression was performed to investigate the extent to which resilience, grit, self-efficacy, self-control, aggression, and life satisfaction could predict mental health disorders. As secondary analyses, independent samples *t*-tests were performed to explore the differences in age, training experience, training frequency, training volume, competitive engagement, mental health disorders, resilience, grit, self-efficacy, self-control, aggression, and life satisfaction between males and females. In addition, Pearson's correlations were conducted between resilience, grit, self-efficacy, self-control, aggression, and mental health disorders. IBM SPSS Statistics (Version 29) was used for data analyses with an alpha level of.05 to determine statistical significance.

## Results

### Demographic, training, and psychological characteristics

Table 1 presents the demographic, training, and psychological characteristics of the participants. Independent samples *t*-tests were performed to explore the differences in age, training experience, training frequency, training volume, competitive engagement, mental health disorders, resilience, grit, self-efficacy, self-control, aggression, and life satisfaction between males and females. Independent samples *t*-tests revealed statistically significant higher training experience *t*(187.461) = 3.015, *p* = .001, Cohen's *d* = .301 (small effect), resilience *t*(418) = 3.216, *p* < .001, Cohen's *d* = .384 (small effect), self-efficacy *t*(418) = 1.708, *p* = .044, Cohen's *d* = .204 (small effect), and aggression *t*(418) = 3.327, *p* < .001, Cohen's *d* = .397 (medium effect) in male than female Brazilian jiu-jitsu athletes.

**Table 1:** Demographic, training, and psychological characteristics.

Characteristics	Male (*n* = 331)	Female (*n* = 89)
Demographic		
Age (SD)	38.45 (8.80)	37.33 (8.62)
Training		
Experience (SD)	6.84 (6.20)*	5.07 (4.51)*
Days/Week (SD)	3.54 (1.35)	3.76 (1.35)
Hours/Week (SD)	6.23 (4.18)	6.08 (3.52)
Competitions (SD)	1.07 (2.01)	1.21 (1.75)
Psychological		
Mental health disorders (SD)	33.46 (10.52)	34.62 (8.80)
Resilience (SD)	3.79 (0.62)*	3.55 (0.68)*
Grit (SD)	3.80 (0.54)	3.81 (0.55)
Self-efficacy (SD)	34.35 (3.70)*	33.60 (3.72)*
Self-control (SD)	46.44 (8.19)	47.15 (7.56)
Aggression (SD)	3.14 (0.89)*	2.79 (0.85)*
Life satisfaction (SD)	26.16 (6.10)	26.36 (5.91)

* *p* < .05; SD, standard deviation.

### Multiple linear regression

Multiple linear regression analyses were conducted to explore the extent to which resilience, grit, self-efficacy, self-control, aggression, and life satisfaction (IVs) could predict mental health disorders in male and female Brazilian jiu-jitsu athletes (Table 2). In male athletes, the multiple linear regression was statistically significant *F*(6,324) = 57.746, *p* < .001. Approximately 51.7% of the variance in mental health disorders was accounted for by its linear relationship with the IVs as a unit. Of the IVs, aggression explained 9.1% (small effect), self-control explained 7.33% (small effect), life satisfaction explained 5.0% (small effect), and resilience explained 1.2% (small effect) of the variance in mental health disorders in male athletes.

**Table 2:** Resilience, grit, self-efficacy, self-control, aggression, and life satisfaction.

Variables			95% CI					
	b	t-value (*p*)	Lower	Upper	sr^2^	F-value (df)	*p*	*R* ^2^
Males								
Mental health disorders						57.746 (6,324)	<.001*	.517
Intercept	56.177							
Resilience	−2.276	−2.819 (.005*)	−3.865	−0.688	−0.0119			
Grit	1.186	1.070 (.286)	−0.996	3.369	0.0017			
Self-efficacy	0.098	0.673 (.501)	−0.188	0.383	0.0007			
Self-control	−0.499	−7.008 (<.001*)	−0.639	−0.359	−0.0733			
Aggression	3.897	7.817 (<.001*)	2.916	4.877	0.0911			
Life-Satisfaction	−0.421	−5.795 (<.001*)	−0.564	−0.278	−0.0501			
Females								
Mental health disorders						11.319 (6,82)	<.001*	.453
Intercept	58.330							
Resilience	−2.598	−1.966 (.053)	−5.227	0.031	−0.0258			
Grit	−1.670	−1.013 (.314)	−4.948	1.608	−0.0069			
Self-efficacy	0.073	0.301 (.764)	−0.411	0.557	0.0006			
Self-control	−0.235	−1.802 (.075)	−0.495	0.025	−0.0217			
Aggression	2.975	2.996 (.004*)	1.000	4.950	0.0599			
Life-Satisfaction	−0.296	−2.127 (.036*)	−0.572	−0.019	−0.0302			

*b*, unstandardized coefficient; sr², semi-partial correlation. *p < .05

In female athletes, the multiple linear regression was statistically significant *F*(6,82) = 11.319, *p* < .001. Approximately 45.3% of the variance in mental health disorders was accounted for by its linear relationship with the IVs as a unit. Of the IVs, aggression explained 6.0% (small effect), and life satisfaction explained 3.0% (small effect) of the variance in mental health disorders in female athletes.

### Correlation matrix

Table 3 presents the zero-sum correlation matrix among the psychological variables explored in this study. In male athletes, significant negative correlations were found between mental health disorder and self-control (*p* < .001, *r* = −.564; large effect), life satisfaction (*p* < .001, *r* = −.436; medium effect), resilience (*p* < .001, *r* = −.385; medium effect), grit (*p* < .001, *r* = −.379; medium effect), and self-efficacy (*p* < .001, *r* = −.331; medium effect). In addition, a significant positive correlation was found between mental health disorders and aggression (*p* < .001, *r* = .529; large effect).

**Table 3:** Zero-Sum correlation matrix.

Variables	1	2	3	4	5	6
Males (*n* = 331)						
1. Resilience	–					
2. Grit	.474*	–				
3. Self-Efficacy	.518*	.555*	–			
4. Self-Control	.369*	.664*	.505*	–		
5. Aggression	−.247*	−.177*	−.172*	−.341*	–	
6. Life Satisfaction	.297*	.322*	.316*	.291*	−.209*	–
7. Mental Health Disorder	−.385*	−.379*	−.331*	−.564*	.529*	−.436*
Female (*n* = 89)						
1. Resilience	–					
2. Grit	.402*	–				
3. Self-Efficacy	.524*	.414*	–			
4. Self-Control	.313*	.535*	.409*	–		
5. Aggression	−.245**	−.246**	−.259**	−.505*	–	
6. Life Satisfaction	.400*	.293*	.377*	.356*	−.217**	–
7. Mental Health Disorder	−.438*	−.408*	−.349*	−.523*	.498*	−.431*

* *p* < .001; **p < .05.

In female athletes, significant negative correlations were found between mental health disorder and self-control (*p* < .001, *r* = −.523; large effect), resilience (*p* < .001, *r* = −.438; medium effect), life satisfaction (*p* < .001, *r* = −.431; medium effect), grit (*p* < .001, *r* = −.408; medium effect), and self-efficacy (*p* < .001, *r* = −.349; medium effect). In addition, a significant positive correlation was found between mental health disorders and aggression (*p* < .001, *r* = .498; medium effect).

## Discussion

This study aimed to examine the extent to which resilience, grit, self-efficacy, self-control, aggression, and life satisfaction, could predict mental health disorders in Brazilian jiu-jitsu athletes.

The current results showed a significant positive relationship between aggression and mental health disorders in male and female athletes, with more aggressive athletes presenting higher mental health disorders. Aggression has been identified as a complex topic, with some authors emphasizing its negative side (28, 29) and others highlighting its positive application (30). Its complexity is exemplified by the multifaceted characteristics of aggression. By definition, aggression represents individual differences in thoughts (hostility), behaviors (verbal and physical), and emotions (anger) intended to harm another person (25). Similarly, previous studies have found that higher aggression significantly correlated with lower mental health (28, 31). In Brazilian jiu-jitsu, athletes are often encouraged to adopt an offensive and aggressive approach while emotionally primed to impose their strategies, dominate their opponents, or win their matches. Despite this encouragement, levels of aggression were found to be similar across all Brazilian jiu-jitsu belt ranks (white, blue, purple, brown, and black), with no significant differences found in total aggression, physical aggression, anger, verbal aggression, and hostility (9). In fact, Blomqvist-Mickelsson (16) found a decrease in aggression after five months of Brazilian jiu-jitsu training in teenager and adult practitioners. In judo, no significant difference in aggression was found between competitors and non-competitors (10). Although previous studies have pointed toward positive effects of Brazilian jiu-jitsu on aggression or no negative associations between aggression and belt ranks or competitive statuses, the current results suggest that the emphasis on aggression should be carefully and examined case-by-case, considering that higher aggression was associated with higher mental health disorder in males and females.

In the current study, results showed a significant negative relationship between self-control and mental health disorders in male athletes, with higher self-control explaining lower mental health disorders. Challenges with self-control are characteristic of several mental disorders presented in the Diagnostic and Statistical Manual of Mental Disorders (24). This finding is convergent with the existing literature, where higher self-control correlated with fewer reports of psychopathology and alcohol abuse (24). He et al. (32) found negative correlations between self-control and symptoms of irritability, depression, and anxiety in university students. Adolescents with lower self-control were also found to be more likely to have higher mental health challenges (33). Simultaneously to the development of offensive and aggressive strategies in Brazilian jiu-jitsu, athletes are expected to abide by several sportsman-like rules. For instance, once an opponent “taps-out” in a submission (e.g., choke or armlocks), one must immediately release the technique to guarantee the opponent's safety. Despite the emotional changes involved during high intensity combative matches, athletes are expected to demonstrate restraint and self-control. These expectations were noted by Blomqvist-Mickelsson (16), who found that five months of Brazilian jiu-jitsu training increased self-control in teenagers and adult practitioners. In addition, a previous study found that higher Brazilian jiu-jitsu training experience positively correlated with self-control (10). Therefore, self-control is not only associated with lower mental health disorders, as found in the current study, but it is also a critical variable to control aggression in the sport domain (34).

The present study revealed a significant negative relationship between life satisfaction and mental health disorders in males and females, with higher life satisfaction explaining lower mental health disorders. Life satisfaction has been discussed as a protective factor against emotional, cognitive, and behavioral disorders, making it an important cognitive indicator of well-being (35). These findings are similar to previous studies who showed a positive correlation between life satisfaction and better mental health (36) and a negative correlation between life satisfaction and anxiety/depression in adults (37). Some authors discuss that the social and mental health benefits of sports may exceed those obtained through alternative leisure activities (5, 6). More experienced Brazilian jiu-jitsu athletes were more likely to present higher life satisfaction than less experienced practitioners (10). In judo, no differences were found in life satisfaction between competitors and non-competitors, suggesting that the recreational practice of combat sports may provide no added benefit to the competition-oriented practice (9).

The current results showed a significant negative relationship between resilience and mental health disorders in male athletes, with higher resilience explaining lower mental health disorders. It has been suggested that resilience is a critical buffer against anxiety and depression (38). More experienced Brazilian jiu-jitsu athletes reported to be more likely to present higher resilience (10). In judo, no differences in resilience were found between competitors and non-competitors (9). Similarly, Ma et al. (39) found higher resilience to correlate with better mental health (emotional, social, and psychological well-being). In addition, a systematic literature review of studies with children and adolescents has unanimously shown higher resilience to be associated with fewer mental health challenges (40).

This study has some limitations. First, the current cross-sectional design prevents causal observations. Second, despite the participants’ anonymity, the use of self-reported instruments may result in social desirability bias. Third, the geographic limitation to the United States may limit the cross-cultural generalizability of the findings. In addition, this study has some strengths and practical applications. First, the reliance on data from participants from different Brazilian jiu-jitsu schools across the United States reflect the association with individuals practicing the combat sport rather than the potential effect of specific teaching methodologies. Second, the biological sex division on the multiple regression analyses showcase the specificities of each group. Third, the results provide practical evidence to inform clinical treatment plans.

The authors recommend future studies to address in a longitudinal design the impact of Brazilian jiu-jitsu on resilience, grit, self-efficacy, self-control, aggression, life satisfaction, and mental health disorders to further advance the limited existing literature. Moreover, future studies are encouraged to explore the mediating and moderating roles of training characteristics such as belt rank, training experience, training frequency, training volume, and competitive status.

The current findings have substantial practical implications and may serve as foundational knowledge for clinical professionals when developing treatment plans to address mental health disorders in Brazilian jiu-jitsu athletes.

In summary, the current findings suggest that aggression, self-control, life satisfaction, and resilience statistically and practically significantly predicted mental health disorders in male Brazilian jiu-jitsu practitioners. Aggression and life satisfaction statistically and practically predicted mental health disorders in female Brazilian jiu-jitsu practitioners. Male athletes presenting higher resilience, self-control, and life satisfaction and lower aggression, and female athletes presenting higher life satisfaction and lower aggression are more likely to present lower mental health disorders than individuals with opposing characteristics. In the current sample, males reported higher training experience, resilience, self-efficacy and aggression than females.

## Data Availability

The raw data supporting the conclusions of this article will be made available by the authors, without undue reservation.
